# Insights into comparative genomics, structural features, and phylogenetic relationship of species from Eurasian *Aster* and its related genera (Asteraceae: Astereae) based on complete chloroplast genome

**DOI:** 10.3389/fpls.2024.1367132

**Published:** 2024-04-26

**Authors:** Hui Chen, Tingyu Li, Xinyu Chen, Tianmeng Qu, Xinyi Zheng, Junjia Luo, Bo Li, Guojin Zhang, Zhixi Fu

**Affiliations:** ^1^ Key Laboratory of Land Resources Evaluation and Monitoring in Southwest, Sichuan Normal University, Ministry of Education, Chengdu, China; ^2^ College of Life Sciences, Sichuan Normal University, Chengdu, China; ^3^ Sichuan Environmental Monitoring Center, Chengdu, China; ^4^ College of Life Sciences, Hunan Normal University, Changsha, China; ^5^ Sustainable Development Research Center of Resources and Environment of Western Sichuan, Sichuan Normal University, Chengdu, China

**Keywords:** *Aster*, chloroplast genome, comparative analysis, Astereae, phylogenetic relationship, divergence time

## Abstract

*Aster* L. is an economically and phylogenetically important genus in the tribe Astereae. Here, the complete plastomes of the eight *Aster* species were assembled and characterized using next-generation sequencing datasets. The results indicated the complete plastomes of *Aster* had a quadripartite structure. These genomes were 152,045–152,729 bp in length and contained 132–133 genes, including 87 protein-coding genes, 37–38 tRNA genes, and eight rRNA genes. Expansion or contraction of inverted repeat regions and forward, palindromic, complement, and reverse repeats were detected in the eight *Aster* species. Additionally, our analyses showed the richest type of simple sequence repeats was A/T mononucleotides, and 14 highly variable regions were discovered by analyzing the border regions, sequence divergence, and hotspots. Phylogenetic analyses indicated that 27 species in Astereae were clustered into six clades, i.e., A to D, North American, and outgroup clades, and supported that the genera *Heteropappus*, *Kalimeris*, and *Heteroplexis* are nested within *Aster*. The results indicated the clades B to D might be considered as genera. Divergence time estimate showed the clades A, B, C, and D diverged at 23.15 Mya, 15.13 Mya, 24.29 Mya, and 21.66 Mya, respectively. These results shed light on the phylogenetic relationships of *Aster* and provided new information on species identification of *Aster* and its related genera.

## 1 Introduction

The tribe Astereaehas has ~222 genera and ~3,100 species, which is the second largest tribe of Asteraceae (Noyes and Rieseberg, 1999; Brouillet et al., 2004; Panero et al., 2004; Panero and Crozier, 2016; Fu et al., 2016). The tribe Senecioneae has over 150 genera and 3,500 species (Nordenstam, 2007), more than the species number of the tribe Astereae. *Aster* is one of the large genera of Astereae and contains more than 152 species. The majority of *Aster* species are distributed in Eurasia, with only one species reaching North America (Nesom, 1994a, b; Chen et al., 2011). The species of *Aster* are mainly perennial herbs and are rarely annual or biennial herbs, subshrubs, or shrubs. The genus is characterized by capitula solitary or arranged in corymbiform or, sometimes, paniculiform capitulescences; white, pink, purple, or blue ray florets; and phyllaries imbricate or arranged in two equal layers.

Traditionally, *Aster* was defined as a genus encompassing around 300 species distributed in both the New World and the Old World (Jones, 1980; Semple and Brouillet, 1980; Jones and Young, 1983). However, in recent years, studies on the basis of morphology (Nesom, 1994a; Nesom and Robinson, 2007), Restriction Fragment Length Polymorphism (RFLPs) (Xiang and Semple, 1994), or DNA markers (Noyes and Rieseberg, 1999; Selliah and Brouillet, 2008; Li et al., 2012; Jafari et al., 2015; Korolyuk et al., 2015) have shown that the New World *Aster* species were distinct from the Old World taxa with a considerable genetic divergence. These New World taxa were treated as 13 separate genera (Nesom, 1994a, b), and the generic delimitation of the Old World species remained controversial. Some studies accepted a border *Aster* s.l., which includes most or all of the species of *Aster* from the Old World (Merxmüller et al., 1976; Ito et al., 1995; Chen et al., 2011). On the contrary, other studies treated the Old World species into *Aster* s.s. and 12 segregated genera (e.g., *Kalimeris* Cass., *Heteroplexis* C.C.Chang, *Heteropappus* Less.) (Tamamschjan, 1959; Grierson, 1975; Nesom, 1994b; Nesom and Robinson, 2007; Chen et al., 2011). However, recent molecular phylogenetic analyses have suggested that neither *Aster* s.l. nor *Aster* s.s. was monophyletic (Selliah and Brouillet, 2008; Pelser et al., 2010; Li et al., 2012; Jafari et al., 2015; Korolyuk et al., 2015; Fu et al., 2019). On the basis of analyses using Internal Transcribed Spacer (ITS), Enternal Transcribed Spacer (ETS), and *trnL-F* sequences, Li et al. (Li et al., 2012). showed that Eurasian *Aster* (referred to as EA *Aster* hereafter) is polyphyletic and supported that the genera *Kalimeris* and *Heteropappus* belonged to *Aster*. In addition, Li et al. (Li et al., 2012). proposed and suggested that *Aster* section *Alpigenia*, *Aster* ser. *Albescentes*, and *Aster* ser. *Hersileoides* should be elevated to the generic rank. However, the taxonomic position of *Aster pycnophyllus* Franch. ex Diels. remained unresolved. Another phylogenetic study using ITS and *psbA*-*trnH* sequences showed that the genera of *Heteropappus* and *Kalimeris* were nested within *Aster*, supporting the results of Jafari et al. (Jafari et al., 2015). Korolyuk et al. (Korolyuk et al., 2015). divided the Eurasian (EA) *Aster* into three groups, namely, a typical Eurasian asters group, *Heteropappus* group, and *Asterothamnus* group, but the relationships among these three groups were not strongly supported, and, hence, the boundary of *Aster* remained unclear. Although, the previous studies have indicated that the non-monophyly of *Aster*, the insufficient sampling of species, and low coverage and inadequate informative sites of molecular markers hampered the resolution of the phylogenetic trees of *Aster* and its related genera.

The chloroplast genome is one of the three DNA genomes, alongside the nuclear and mitochondrial genomes. In general, it is inherited maternally and possesses a highly conserved circular DNA arrangement, typically ranging from 115 kb to 165 kb in size (Wicke et al., 2011; Daniell et al., 2016). The complete chloroplast genome is a quadripartite structure, consisting of a large single copy (LSC), a small single copy (SSC), and two inverted repeats (IRs) (Daniell et al., 2016). The length differences are mostly due to expansion/contraction of IR regions (Zhu et al., 2016) or gene losses (Magee et al., 2010). In addition, the complete sequences of chloroplast genomes are commonly used for phylogenetic reconstruction at lower taxonomic levels, e.g., within genus, and population genetic analyses in plants. The utilization of complete chloroplast genomes has become widespread as an efficient tool for molecular phylogenetics in *Aster* (Kumar et al., 2009; Choi and Park, 2015; Zhang et al., 2015; Shen et al., 2017; Wang et al., 2019; Zhang et al., 2019a; Zhang X. et al., 2021; Duan et al., 2022; Palazzesi et al., 2022; Wang and Liu, 2023) and other tribes of Asteraceeae (Vargas et al., 2017; Do et al., 2019; Tyagi et al., 2019; Zhang et al., 2019b; Yu et al., 2022; Liu et al., 2023). Previous studies on *Aster* classification used one to several molecular markers, such as ITS, ETS, *trnL-F*, and *psbA-trnH* sequences (Li et al., 2012; Jafari et al., 2015), and some studies used only ITS sequences (Korolyuk et al., 2015), leaving many phylogenetic and taxonomic questions unresolved. Additionally, the lack of complete chloroplast genome sequences severely hampers the evaluation analyses of the genetic diversity of *Aster* germplasm resources.

In this study, to explore the genetic variation of *Aster* and its related genera, we report eight newly sequenced chloroplast genomes in the genus *Aster*, namely, *Aster polius* C.K. Schneid., *Aster albescens* Wall., *Aster argyropholis* Hand.-Mazz., *Aster lavandulifolius* Hand.-Mazz., *Aster procerus* Hemsl., *A. pycnophyllus*, *Aster falcifolius* Hand.-Mazz., and *Aster yunnanensis* Franch. The objectives of this study were to (1) analyze the evolution of chloroplast genomes within *Aster* using genetic comparative methods, (2) reconstruct the phylogenetic relationships of *Aster* and its related genera and further determine the phylogenetic backbone of *Aster*, and (3) estimate the divergence time of *Aster* and its related genera. This study provides new insights into the phylogenetics and evolution of *Aster* and its related genera and also shed the lights on the genetic diversity of *Aster* wild germplasm resources.

## 2 Materials and methods

### 2.1 Sampling, extraction, and genome sequencing

Fresh leaves of the eight *Aster* species were gathered from the wild (**Table 1**). The formal identification of the plant material was undertaken by Dr. Zhixi Fu. The voucher specimens were then preserved in the herbarium at Sichuan Normal University in China (SCNU) (contact person: Dr. Zhixi Fu, fuzx2017@sicnu.edu.cn). As these species are not included in List of National Key Protected Wild Plants in China, there was no need to obtain a permit for their collection. Following the CTAB DNA extraction protocol (Allen et al., 2006), genomic DNA was extracted using the Plant Genomic DNA Kit (Tiangen, Beijing, China). The construction of the DNA library was carried out using the Illumina Paired-End DNA Library Kit (Illumina Inc., San Diego, CA, USA), and, subsequently, sequencing was performed on the Illumina Genome Analyzer (Hiseq 2000, Illumina, San Diego, CA, USA). The resulting raw data for each of the eight species consisted of approximately 150-bp paired-end read lengths. The 27 complete chloroplast genomic datasets are available for download on NCBI (**Table 2**). In the final supermatrix, names of species were checked based on Flora of China (Chen et al., 2011).

**Table 1:** Information on the 27 *Aster* species used in the study.

Species	GenBank	Voucher no.	Locality information of newly sequenced species
*Aster altaicus*	NC034996.1	/	/
*Aster ageratoides*	MW813970.1	/	/
*Aster albescens*	OM912718.1	FZX 2899	Li county, Sichuan province
*Aster argyropholis*	OM912719.1	FZX 2970	Jinchuan county, Sichuan province
*Aster batangensis*	MZ292735.1		
*Aster falcifolius*	ON515469.1	FZX 4120	Mao county, Sichuan province
*Aster fanjingshanicus*	ON055287.1	/	/
*Aster flaccidus*	MN122101.1	/	/
*Aster hersileoides*	NC042944.1	/	/
*Aster hypoleucus*	NC046503.1	/	/
*Aster indicus*	MG710386.1	/	/
*Aster lavanduliifolius*	OM912720.1	FZX 4049	Jinchuan county, Sichuan province
*Aster pekinensis*	MW255593.1	/	/
*Aster polius*	OM912721.1	FZX 2922	Xiaojin county, Sichuan province
*Aster procerus*	ON515467.1	FZX 693	Linan city, Zhejiang province
*Aster pycnophyllus*	ON515468.1	FZX 4080	Dali city, Yunnan province
*Aster souliei*	OK323961.1	/	/
*Aster spathulifolius*	NC 027434.1	/	/
*Aster tataricus*	NC 042913.1	/	/
*Aster tongolensis*	OK323962.1	/	/
*Aster yunnanensis*	ON515470.1	FZX 241	Fugong county, Yunnan province
*Heteroplexis incana*	NC 048508.1	/	/
*Heteroplexis sericophylla*	MK942054.1	/	/

**Table 2:** Comparative analysis of chloroplast genomes of the seven *Aster* species.

Species	*Aster polius*	*Aster albescens*	*Aster argyropholis*	*Aster lavandulifolius*	*Aster procerus*	*Aster falcifolius*	*Aster pycnophyllus*	*Aster yunnanensis*
GenBank accession	OM912721	OM912718	OM912719	OM912720	ON515467	ON515469	ON515468	ON515470
Plastome size (bp)	152,045	152,729	152,725	152,719	152,656	152,664	152,721	152,589
LSC length (bp)	83,716	84,410	84,405	84,399	84,438	84,386	84,470	84,374
IR length (bp)	25,046	25,055	25,055	25,055	24,980	25,040	24,988	25,025
SSC length (bp)	18,237	18,209	18,210	18,210	18,258	18,198	18,275	18,165
GC content (%)	37.35	37.3	37.29	37.29	37.27	37.3	37.28	37.31
Number of genes	133	133	133	133	132	133	132	133
Protein-coding genes	87	87	87	87	87	87	87	87
tRNA genes	38	38	38	38	37	38	37	38
rRNA genes	8	8	8	8	8	8	8	8

### 2.2 Assembly and annotation of chloroplast genome

For the assembly of the chloroplast genome, the software SPAdes 3.15.1-Linux was employed, utilizing the default parameters (Prjibelski et al., 2020). To evaluate the assembly quality, the circular maps were identified using Bandage software (Wick et al., 2015). Subsequently, the resulting assembly was annotated using PGA (Qu et al., 2019), referencing the chloroplast genome sequence of *Eschenbachia blinii* (H.Lév.) (NC 037605.1). The annotation results were then checked using Geneious R11 (Kearse et al., 2012). The chloroplast genome map was visualized using OGDRAW (https://chlorobox.mpimp-golm.mpg.de/OGDraw.html). Additionally, the tRNA sequences were validated using tRNAscan-SE v2.0 (Chan et al., 2021), available on the Geseq platform (https://chlorobox.mpimp-golm.mpg.de/geseq.html). The annotated chloroplast genomes have been submitted to GenBank (**Table 2**). Analysis of plastid information was conducted using Geneious R11.

### 2.3 Comparative genome analysis

To identify potential IR expansion or contraction in eight *Aster* species, the reference species was used from *A. ageratoides*. This analysis was conducted using the perl script of Irscope (Amiryousefi et al., 2018). With the *A. albescens* as reference, the homology of these sequences was visualized using the mVISTA program (Frazer et al., 2004, https://genome.lbl.gov/vista/mvista/submit.shtml) with the LAGAN mode (Brudno et al., 2003).

### 2.4 Repeat sequences and SSR analysis

In this study, the identification of direct (forward), inverted (palindromic), complement, and reverse repeats elements was identified by REPuter (Kurtz et al., 2001), with maximum computed repeats equal to 50 bp, hamming distance of 3, and minimal repeat size of 30 bp. Furthermore, the detection of simple sequence repeats (SSRs) within the complete chloroplast genomes was performed using Microsatellite (MISA) (Beier et al., 2017). The thresholds for SSR detection were set to 10, 5, 4, 3, 3, and 3, for mono-, di-, tri-, tetra-, penta-, and hexa-nucleotides, respectively.

The alignment of all sequences from the eight *Aster* species was performed using the “–auto” strategy of Multiple Alignment using Fast Fourier Transform (MAFFT). Nucleotide diversity was then calculated using a sliding window approach in DnaSP v.6.12.03 (Rozas et al., 2017) with a window length of 600 bp and a step size of 200 bp.

### 2.5 Codon usage analysis

MEGA v 7.0 was used to analyze the synonymous codon usage and the relative synonymous codon usage (RSCU) of the *Aster* cp genomes. RSCU values >1 represent frequently used codons than expected, whereas values <1 signify the opposite. Codons having no preference value are set to 1.00.

### 2.6 Phylogenetic analysis

The phylogenetic analysis of the complete chloroplast genomic dataset, consisting of 27 species of Astereae, was performed using the maximum likelihood (ML) method implemented in RAxML. The species of *Nannoglottis ravida* and *Llerasia caucana* from basal group of Astereae were selected as outgroups (Stamatakis et al., 2008). The analysis was performed on the CIPRES platform (Miller et al., 2010) (https://www.phylo.org/portal2/). ModelTest (Posada, 2006) was employed to determine the most suitable model for the dataset. The molecular model GTRCAT was applied for the analysis. For bootstrap support assessment, the fast bootstrap option with 1,000 replicates was utilized in RAxML from CIPRES platform. The morphological identification characteristics of the genus *Aster* and its related genera have been described more clearly by Chen et al. (2011). Therefore, we define the key to the *Aster* and related species with reference to the criteria proposed by Chen et al. (2011) in combination with classification of previous studies (Li et al., 2012; Jafari et al., 2015; Korolyuk et al., 2015).

### 2.7 Divergence time estimations

For divergence time estimation, we used the complete chloroplast sequence dataset. The BEAST v.1.8 (Drummond et al., 2012) was applied to estimate the divergence times with Bayesian uncorrelated lognormal relaxed clock model. The node of Astereae was set at 31.42 Mya according to Zhang C. F. (Zhang C. F. et al. 2021). The tree Yule model was selected. The Markov chain Monte Carlo (MCMC) was run for 10,000,000 generations and sampled every 1,000 generations. TreeAnnotator v. 1.6 (BEAST package) was used to summarize and annotate the tree, with the initial 10% of trees discarded as burn-in. Finally, the tree was visualized in the program Figtree v.1.4.4 (http://tree.bio.ed.ac.uk/) with 95% highest posterior density being shown.

## 3 Results

### 3.1 Chloroplast genome structure and feature of *Aster*

**Figure 1:**
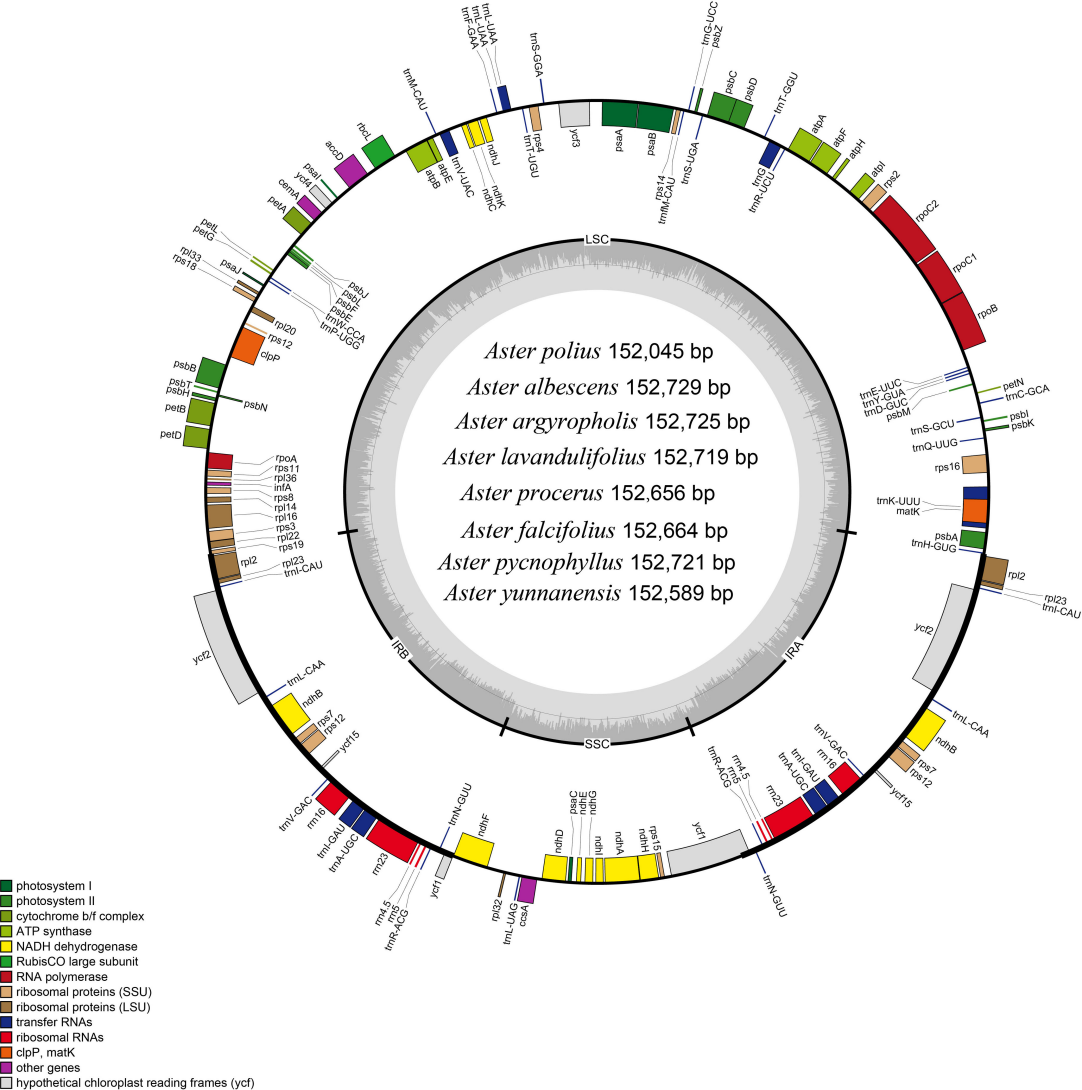
Gene map of the *Aster* chloroplast genome. Genes shown outside the outer circle are transcribed clockwise, and those insides are transcribed counterclockwise. Genes are color-coded according to different functional groups. The darker gray in the inner circle indicates the GC content, and the lighter gray indicates the AT content. The inner circle also shows that the chloroplast genome contains two copies of inverted repeats (IRA and IRB), a large single-copy (LSC) region, and a small single-copy (SSC) region.

In this study, the complete chloroplast genomes of the eight species of *Aster* were sequenced and assembled. The results revealed a high degree of conservation in the structures of these genomes (**Figure 1**). These chloroplast genomes exhibited the standard quadripartite structure, consisting of a LSC region, a SSC region, and a pair of IR regions (IRa and IRb). The size of these genomes varied from 152,045 bp (*A. polius*) to 152,729 bp (*A. albescens*) (**Table 2**). The GC content ranged from 37.27% (*A. procerus*) to 37.35% (*A. polius*). Overall, all chloroplast genomes have 133 genes except *A. procerus* and *A. pycnophyllus* having 132 genes, including 87 protein-coding genes, 37/38 tRNA genes, and eight rRNA genes. Additionally, 115 of these genes were unique and 18 genes were duplicated in the IR regions (**Table 3**). The arrangement of these 133 genes in all chloroplast genomes was found to be completely collinear. There were two introns of four genes (*rps12*, *rps12*, *ycf3*, and *clpP*) and single intron of 16 genes (*ndhA*, *ndhB*, *petB*, *petD*, *atpF*, *rbcL*, *rpl16*, *rpl2*, *rps16*, *rpoC1*, *trnA-UGC*, *trnG*, *trnI-GAU*, *trnK-UUU*, *trnL-UAA*, and *trnV-UAC*). The gene *rps12* was trans-spliced, and the genes *ndhD* and *psbL* experienced RNA editing.

**Table 3:** List of genes found in the complete chloroplast genomes of *Aster* species.

Category	Gene group	Gene name
Photosynthesis	Subunits of photosystem I	*psaA*, *psaB*, *psaC*, *psaI*, *psaJ*
	Subunits of photosystem II	*psbA*, *psbB*, *psbC*, *psbD*, *psbE*, *psbF*, *psbH*, *psbI*, *psbJ*, *psbK*, *psbL*, *psbM*, *psbN*, *psbT*, *psbZ*
	Subunits of Nicotinamide adenine dinucleotide (NADH) dehydrogenase	ndhA*, ndhB*(2), *ndhC*, *ndhD*, *ndhE*, *ndhF*, *ndhG*, *ndhH*, *ndhI*, *ndhJ*, *ndhK*
	Subunits of cytochrome b/f complex	*petA*, petB*, petD*, *petG*, *petL*, *petN*
	Subunits of ATP synthase	*atpA*, *atpB*, *atpE*, atpF*, *atpH*, *atpI*
	Large subunit of rubisco	rbcL*
Self-replication	Proteins of large ribosomal subunit	*rpl14*, rpl16*, rpl2*(2), *rpl20*, *rpl22*, *rpl23(2)*, *rpl32*, *rpl33*, *rpl36*
	Proteins of small ribosomal subunit	*rps11*, rps12**(2), *rps14*, *rps15*, rps16**, *rps18*, *rps19*, *rps2*, *rps3*, *rps4*, *rps7(2)*, *rps8*
	Subunits of RNA polymerase	*rpoA*, *rpoB*, rpoC1*, *rpoC2*
	Ribosomal RNAs	*rrn16(2)*, *rrn23(2)*, *rrn4.5(2)*, *rrn5(2)*
	Transfer RNAs	trnA-UGC*(2), *trnC-GCA*, *trnD-GUC*, *trnE-UUC*, *trnF-GAA*, trnG*, *trnG-UCC*, *trnH-GUG*, *trnI-CAU(2)*, trnI-GAU*(2), trnK-UUU*, *trnL-CAA(2)*, *trnL-UAA*, trnL-UAA*, *trnL-UAG*, *trnM-CAU*, *trnN-GUU(2)*, *trnP-UGG*, *trnQ-UUG*, *trnR-ACG(2)*, *trnR-UCU*, *trnS-GCU*, *trnS-GGA*, *trnS-UGA*, *trnT-GGU*, *trnT-UGU*, *trnV-GAC(2)*, trnV-UAC*, *trnW-CCA*, *trnY-GUA*, *trnfM-CAU*
Other genes	Maturase	*matK*
	Protease	clpP**
	Envelope membrane protein	*cemA*
	Acetyl-CoA carboxylase	*accD*
	c-Type cytochrome synthesis gene	*ccsA*
	Translation initiation factor	*infA*
Genes of unknown function	Conserved hypothetical chloroplast open reading frame (ORF)	*#ycf1*, *ycf1*, *ycf15(2)*, *ycf2(2)*, ycf3**, *ycf4*

Gene*, gene with one introns; Gene**, gene with two introns; #Gene, pseudo-gene; Gene(2), number of copies of multi-copy genes.

### 3.2 Expansion and contraction of the border regions

**Figure 2:**
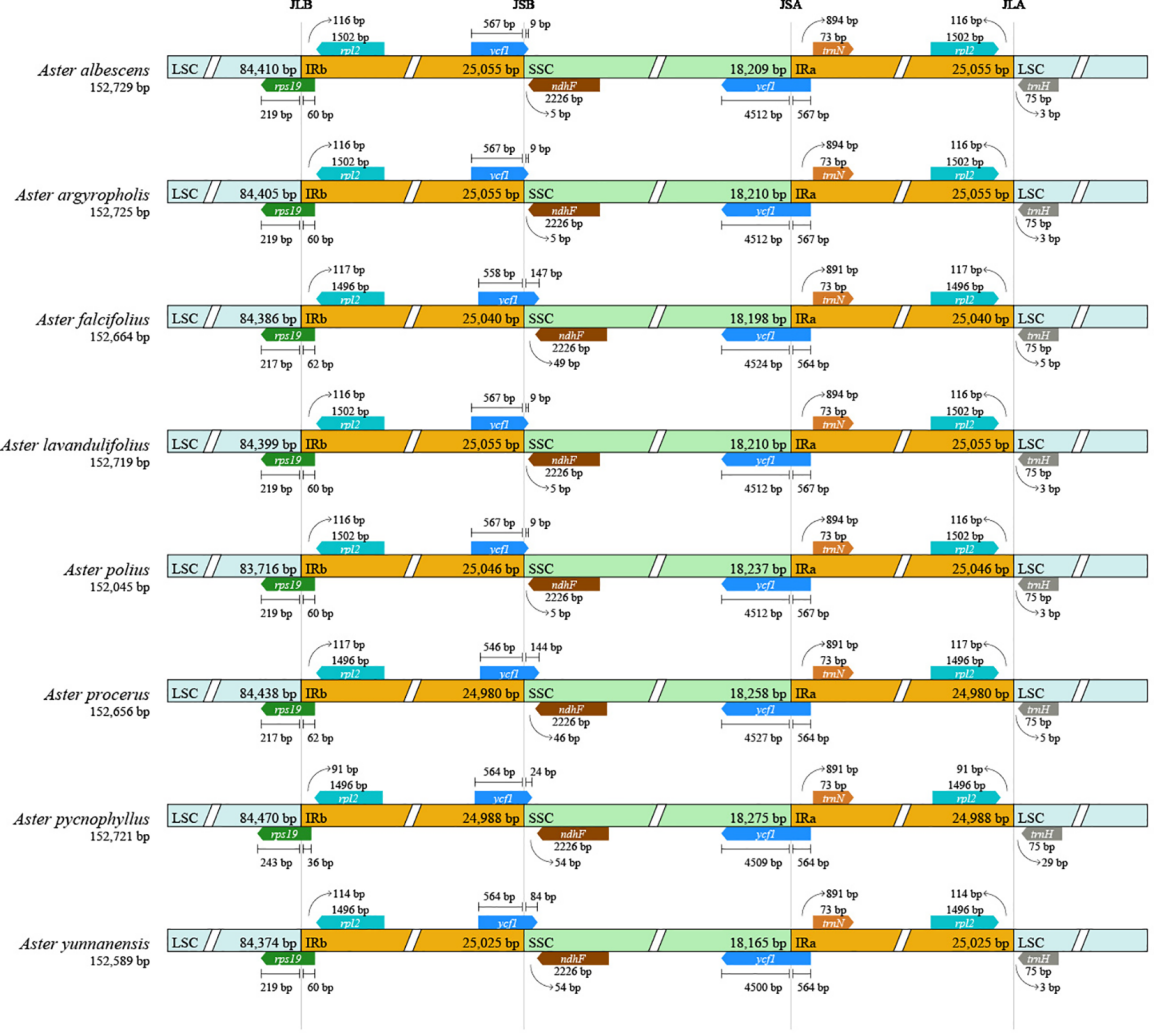
Comparison of IR-SC border positions across plastomes of the eight *Aster* taxa. Genes are denoted by colored boxes. The gaps between the genes and the boundaries are indicated by the base lengths (bp).

In general, the IR/Single Copy (SC) expansion and contraction might cause the IR/SC junction position change. The IR/SC borders of the eight newly sequenced *Aster* chloroplast genomes were compared to analyze the expansion and contraction variation in junction regions (**Figure 2**). Although overall genomic structure including gene order and gene number was well conserved, these genomes exhibited slight differences at four junctions (JLB, JSB, JSA, and JLA). The *rps19* gene of all *Aster* species located the JLBs, with the IRa region including 60 bp to 62 bp, except for *A. pycnophyllus* (36 bp). Likewise, the JLAs of all *Aster* species were located between *rps12* and *trnH*. The *ndhF* gene, related to photosynthesis, was entirely located in the SSC region and the distance to the junction ranged from five to 54 bp. In our newly sequenced genomes, the *ycf1* pseudogene was identified in all newly sequenced genomes. The main part of *ycf1* gene was in the SSC region, with other 564 bp to 567 bp in the IRa region. The same fragment was also found in the IRb region of the *ycf1* pseudogene and extended to SSC region with extension region with 9 bp to 147 bp.

### 3.3 Repeat sequence analysis

**Figure 3:**
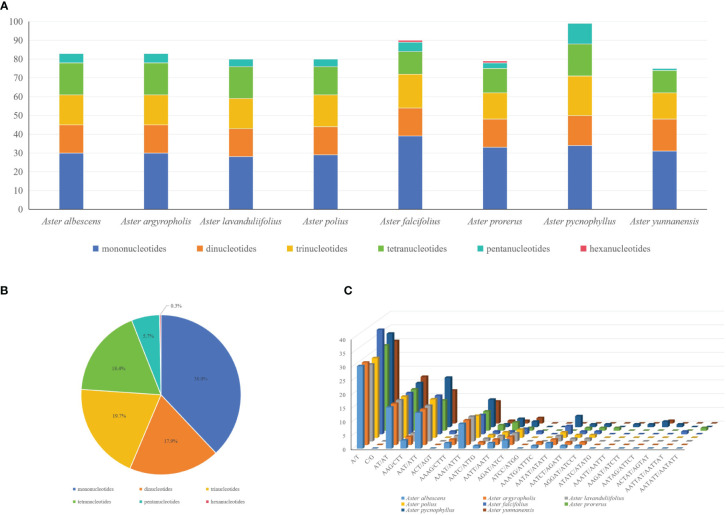
Comparison of simple sequence repeats (SSRs) among eight plastomes. **(A)** Numbers of SSRs detected in the eight newly sequenced *Aster* plastomes. **(B)** Frequencies of identified SSR types in all eight *Aster* plastomes. **(C)** Analysis of SSRs in eight *Aster* plastid genomes species.

In the SSR analysis of the six species of Astereae, 75 (*A. yunnanensis*) to 99 (*A. pycnophyllus*) SSRs were found, showing a similar number of SSRs in Astereae (**Figure 3A**). In addition, these detected SSRs can be divided into six types, including mononucleotides (38%), dinucleotides (18.4%), trinucleotides (19.7%), tetranucleotides (17.9%), pentanucleotides (5.7%), and hexanucleotides (0.3%) (**Figure 3B**). The hexanucleotide repeats were only found in the chloroplast genomes of *A. falcifolius* and *A. prorerus*. The four dominant motif types of these SSRs were A/T (28–38), AT/AT (15–17), AAT/ATT (11–18), and AAAT/ATTT (7–10) (**Figure 3C**).

**Figure 4:**
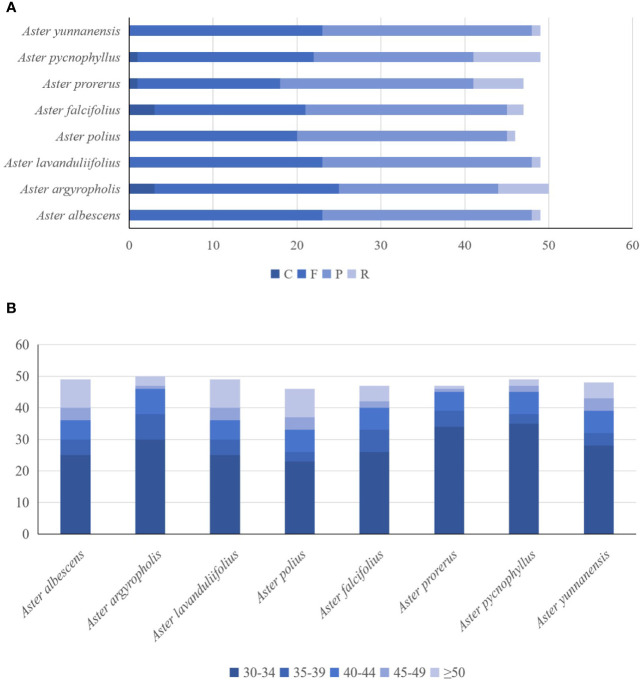
The analysis of the number and length of the long repeats identified from the eight *Aster* complete chloroplast genomes. The hamming distance of 3, the minimal repeats of 30, and the maximum repeats of 5,000 were applied during the calculating process. **(A)** Numbers of the type of long repeats contains F, P, R, and C. **(B)** Numbers of the length of long repeats.

The forward, palindromic, complement, and reverse repeats were detected in the eight newly sequenced chloroplast genomes (**Figure 4A**). *A. procerus*, *A. pycnophyllus*, *A. yunnanensis*, and *A. falcifolius* had all four type repeats. *A. polius*, *A. albescens*, *A. argyropholis*, and *A. lavandulifolius* had forward, palindromic, and reverse repeats. On average, 46–49 repeat sequences were identified in these genomes, with 17–23 forward repeats, 19–25 palindromic repeats, and 1–8 reverse repeats. However, complement repeats were only detected in *A. procerus*, *A. pycnophyllus*, *A. yunnanensis*, and *A. falcifolius*, with number of 1 to 3. Moreover, the repeats with 30 bp to 39 bp in length were the most common type in these genomes (**Figure 4B**), and none of the repeats with 50 bp to 59 bp in length. These interspersed repeat sequences were mainly present in the intergenic spacers, and several were observed within the coding regions and introns. The *ycf15*, *rps12*, *ycf2*, *rrn5*, *rrn4*.5, *psbN*, *trnG*, *trnT*-*GGU*, *ycf4*, *cemA*, *trnS*-*GCU*, *trnS*-*UGA*, *trnS*-*GGA*, *psaB*, *psaA*, *accD*, *psal*, *psbE*, *petL*, *ndhD*, and *psaC* genes contained LDRs. The interspersed repeat sequences were also more commonly detected in LSC and IR than SSC regions. The overall distribution of interspersed repeat sequences was similar in both IR regions.

### 3.4 Sequence divergence and hotspots

**Figure 5:**
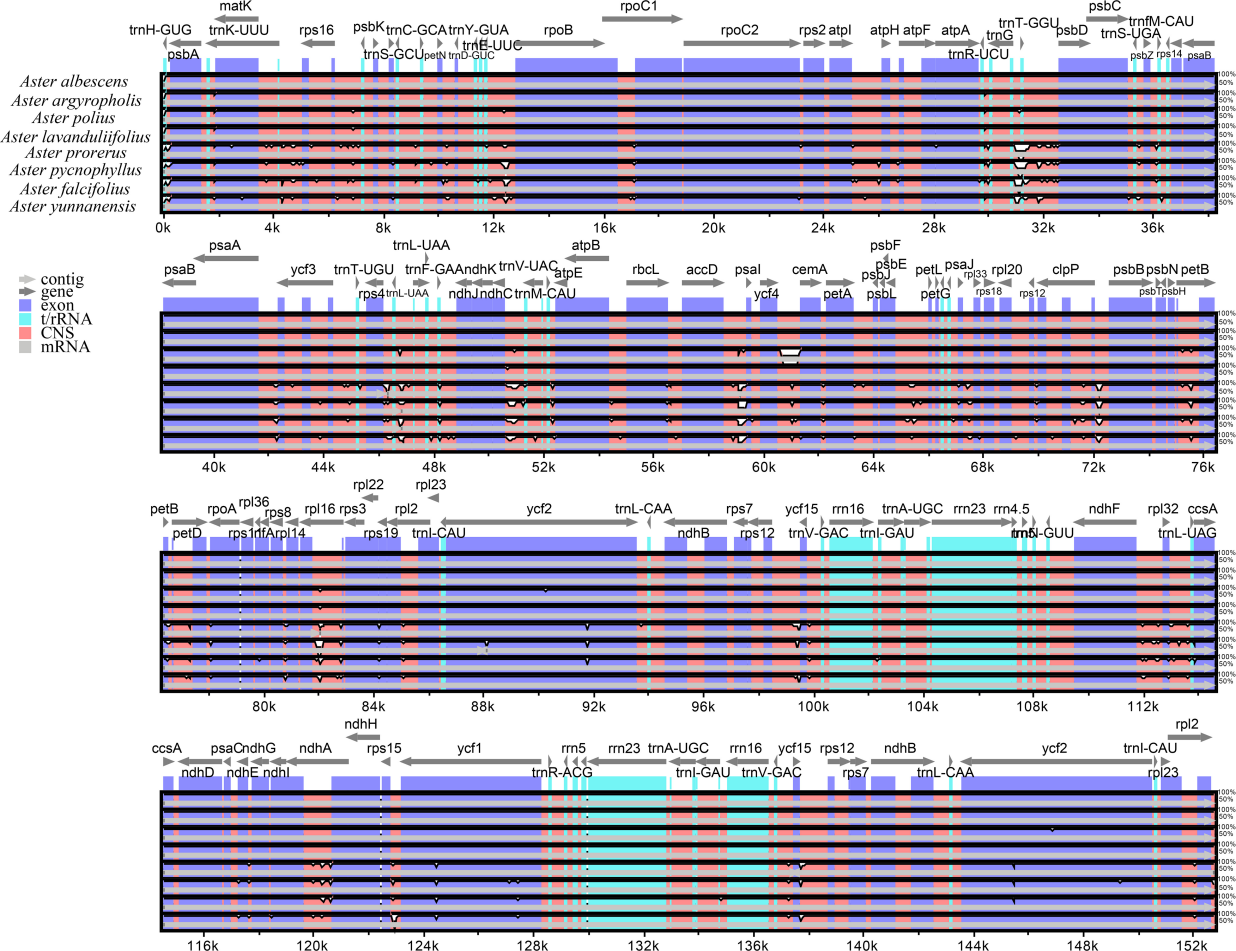
Comparison of the chloroplast genome of the eight *Aster* newly sequenced species. Dark blue bars represent protein-coding genes, pale blue bars represent rRNA genes, and red bars represent conserved non-coding sequences. The y-scale axis represents the percentage identity (50%–100%). mVISTA was used to perform the comparison.

**Figure 6:**
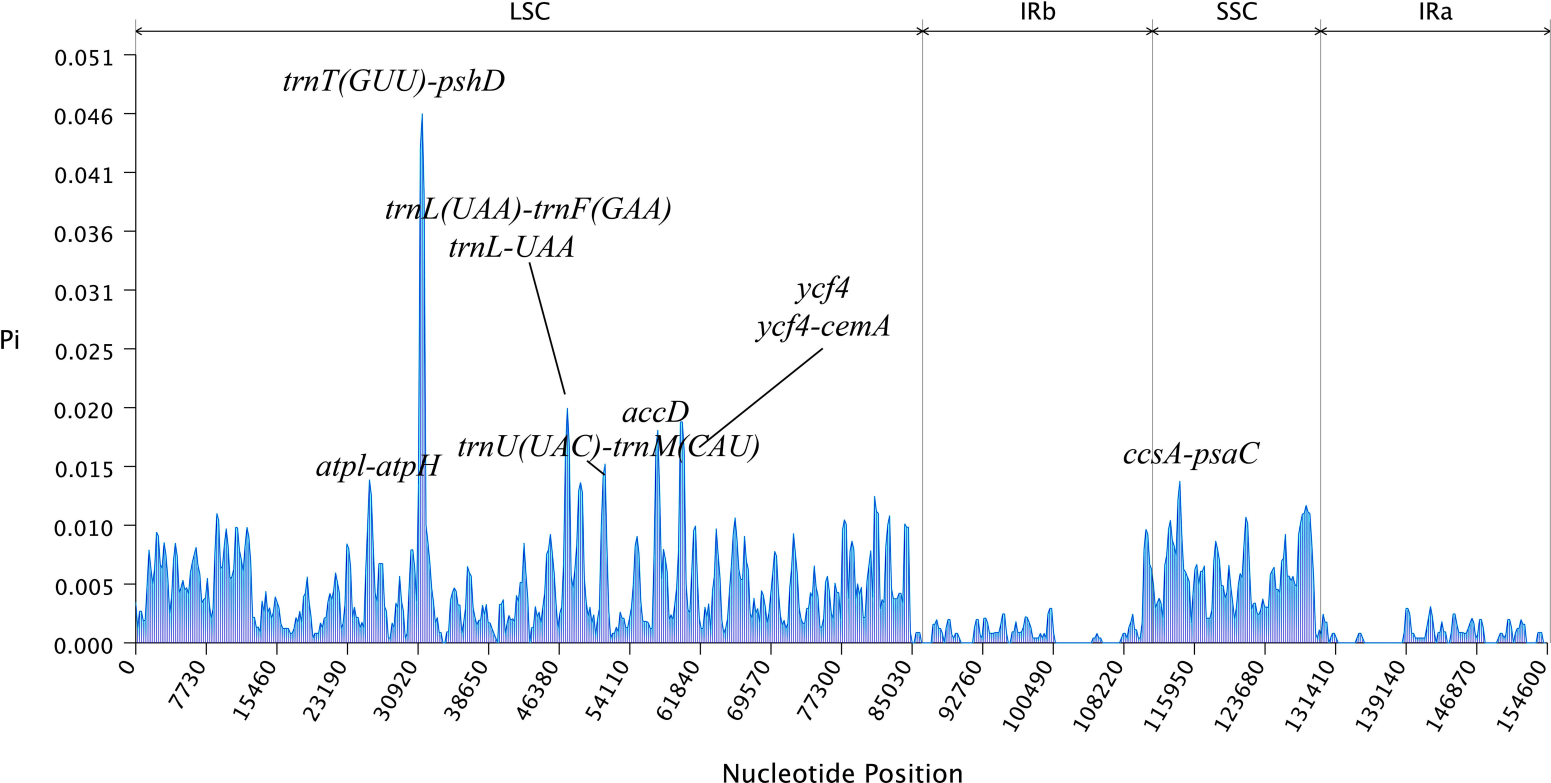
The nucleotide variability (Pi) values were compared among the eight *Aster* taxa.

The mVISTA analysis of these eight *Aster* species indicated the complete chloroplast genome shared high levels of sequence similarity. Genetic variability was more prevalent in the non-coding regions than in the coding regions. The five genes with the highest variation were *matK*, *atpA*, *rps19*, *ycf2*, and *ycf1* (**Figure 5**). DnaSP analysis revealed nucleotide diversity in single copy genes and intergenic regions with nucleotide diversity (Pi) ranged from 0.00068 to 0.04577. Six mutation hotspots showed significantly high Pi values (π > 0.014) (**Figure 6**), much higher than the average pi value (π = 0.0038). Among the gene coding regions, the highest Pi values were found in *ndhF*, followed by *ndhC*, *trnV* (*UAC*), and *trnM (CAU)*. Among intergenic regions, the highest Pi values were detected in the *rpl12*-*ndhF* region, followed by *ndhC*–*trnV* (*UAC*), *trnV* (*UAC*)–*trnM* (*CAU*), and *trnM* (*CAU*)–*atpE* (**Figure 6**). We analyzed the nucleotide diversity of the complete chloroplast genomes and the LSC, SSC, and IR regions. The nucleotide diversity in the complete chloroplast genome was 0.0038, and higher nucleotide diversity was found in the LSC and SSC regions than the IR region, showing that the IR regions were more conserved than the single-copy regions. We found only two regions with π > 0.02, i.e., *trnT* (*GGU*)–*psbD* and *trnL* (*UAA*)–*trnF* (*GAA*), and three regions with π>0.015 and <0.02, i.e., *trnU* (*UAC*)–*trnM* (*CAU*), *accD*, and *ycf4*/*ycf4-cemA* (**Figure 6**).

### 3.5 Codon usage analysis

**Figure 7:**
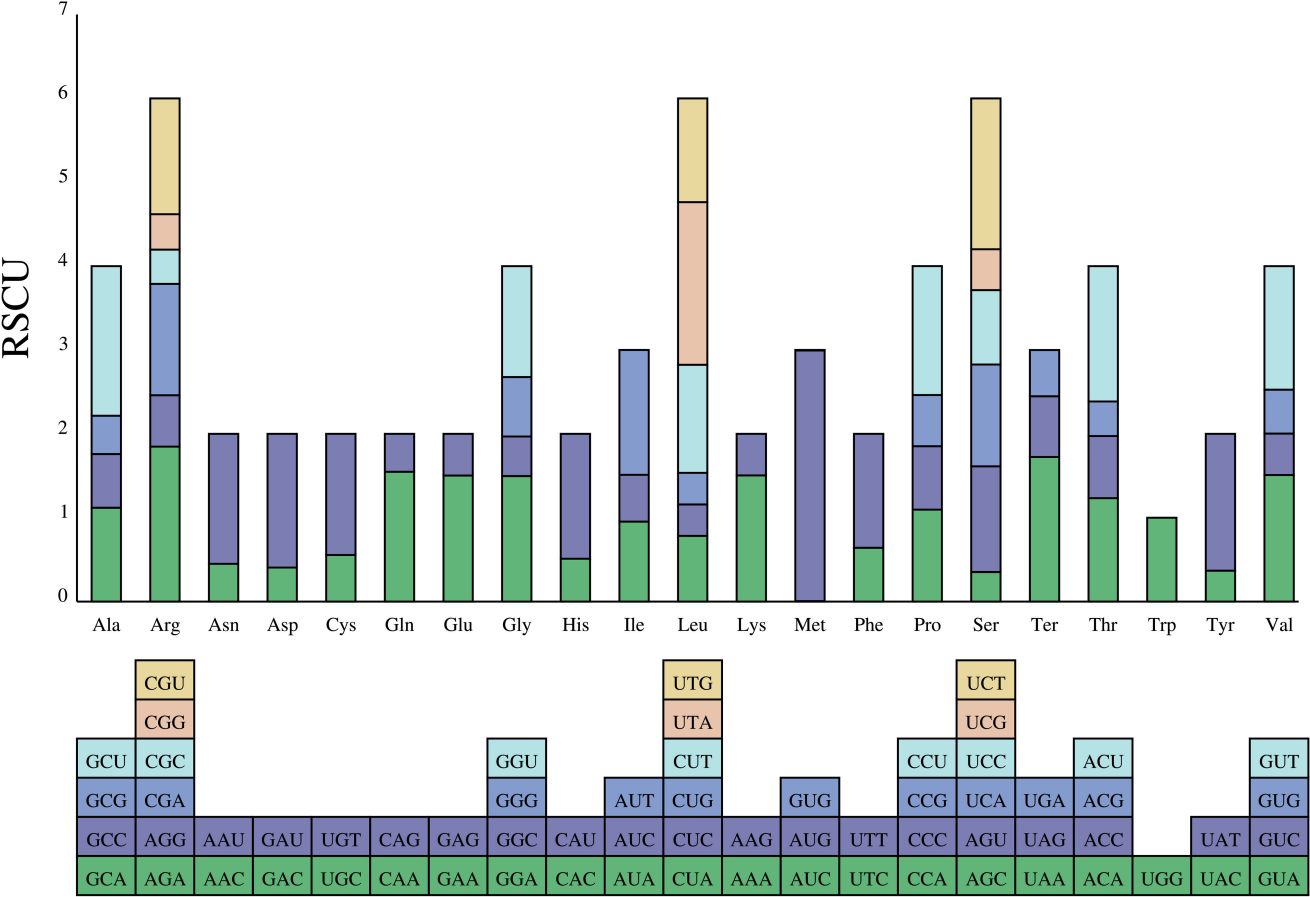
The RSCU values of the 20 amino acids of the complete chloroplast genome of the eight *Aster* taxa and their different codon usages.

The preferences for codon are extremely similar among species. The analyses showed that 87 protein-coding genes were encoded by 64 codons (including three are stop codons: UAA, UGA, and UAG; **Figure 7**). The most prevalent amino acid was leucine. Leucine was encoded by CUA, CUC, CUG, CUU, UUA, and UUG with 2,420 codons (*A. falcifolius*) to 2,440 codons (*A. yunnanensis*). However, the rarest one was cysteine. Cysteine was encoded by UGC and UGU with 251 codons (*A. falcifolius*) *to* 252 codons (*A. polius*). In the complete chloroplast genome of these *Aster*, only codons tryptophan (encoded by UGG) exhibited no bias with RSCU = 1.00. The common start codon for the protein coding genes was AUG (M), except for the *psbL*, *rps19*, and *ndhD* genes, which have start codons of ACG, GUG, and GUG in all species, respectively.

### 3.6 Phylogenetic analysis

**Figure 8:**
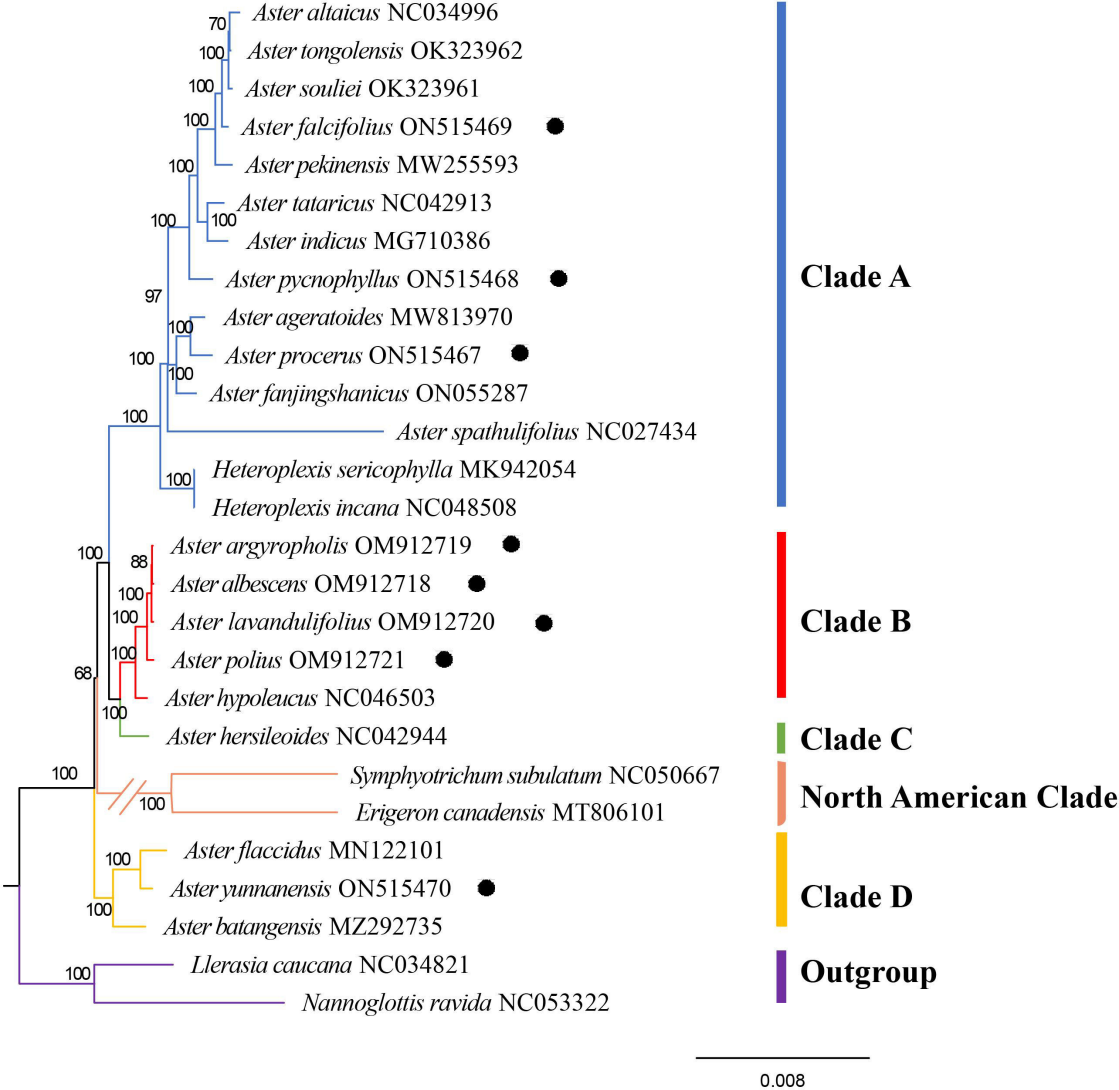
The best maximum likelihood (ML) phylogram inferred from 27 chloroplast genomes (bootstrap value are indicated on the branches). The circled species are the newly sequenced species in this study.

In this study, the complete chloroplast genomes of 27 Astereae species were used to perform phylogenetic reconstruction, with *Nannoglottis ravida* (C.Winkl.) Y.L.Chen and *Llerasia caucana* (S.F.Blake) Cuatrec used as outgroup. Phylogenetic analyses of the supermatrix of 25 taxa (not include outgroups) using the ML methods yielded a topology (**Figure 8**) with in-group fell into five clades: clade A, clade B, clade C, clade D, and North American clade (**Figure 8**). Clade A was the largest clade and was strongly supported (Bootstrap value (BP) = 97/100), containing 12 *Aster* species and two *Heteroplexis* species. The newly sequenced species, namely, *A. falcifolius*, *A. pycnophyllus*, and *A. procerus*, were nested in clade A. Other four newly sequenced species, namely, *A. argyropholis*, *A. albescens*, *A. lavandulifolius*, and *A. polius*, together with *Aster hypoleucus* Hand.-Mazz. formed the strongly supported clade B. *A. hersileoides* lonely formed clade C, as the sister group of clade B with a moderate support (BS = 68). *Symphyotrichum subulatum* (Michx.) G.L. Nesom and *Erigeron canadensis* L. formed the North American clade, and it was a sister group of clades A, B, and C with a high support (BS = 100). The newly sequenced *A. yunnanensis* were placed together with *A. flaccidus* and *A. batangensis* in clade D with strong support (BS = 100).

### 3.7 Divergence time estimations

**Figure 9:**
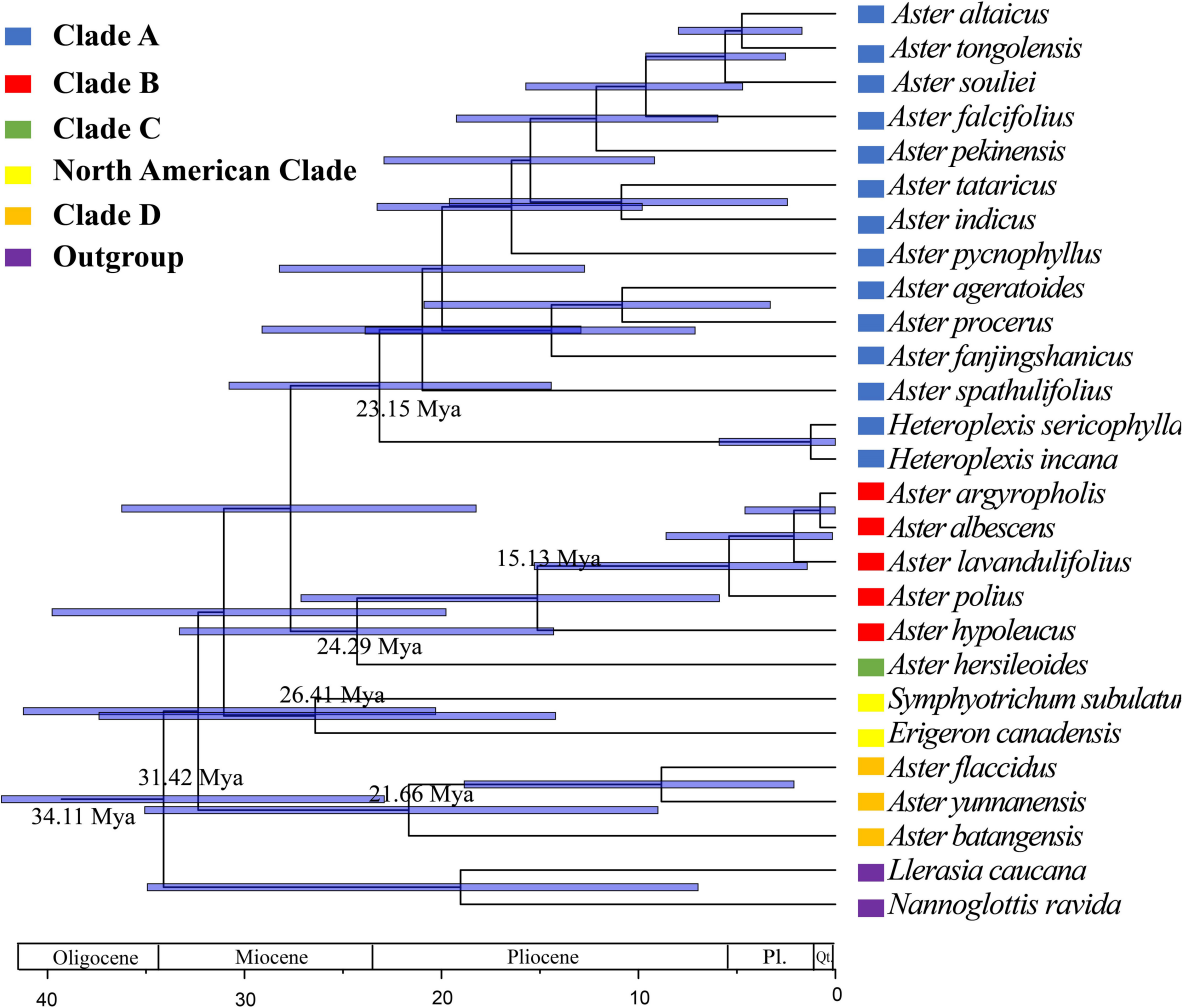
Divergence time estimates of *Aster* based on complete cp genomes, based on BEAST analysis using the complete chloroplast genomes dataset. Blue bars indicate 95% highest posterior density intervals.

On the basis of the newly reconstructed phylogeny, the origin and divergence times of lineages within the genus *Aster* were estimated (**Figure 9**). Divergent time estimate showed that the divergent time of clade A was dated back to 23.15 Mya. clades B, C, and D were divergent from 15.13 Mya, 24.29 Mya, and 21.66 Mya, respectively.

## 4 Discussion

### 4.1 Plastome structure and characteristics analysis

The structure, gene position, size, orientation, and gene content of the plastid genomes of the eight *Aster* species were highly conserved (Doorduin et al., 2011; Curci et al., 2015; Cheon et al., 2017; Chen et al., 2018). These genomes of *Aster* have a standard quadripartite structure, including a LSC, a SSC, and a pair of IRs (IRa and IRb), which was the same as that reported for most other *Aster* (Shen et al., 2018; Wang et al., 2019; Zhang X. et al., 2021). The sizes of plastomes of the eight *Aster* species are between 152,045 bp and 152,729 bp (**Table 2**). In addition, these plastomes did not have any loss of introns. Additionally, the GC contents, which are a crucial factor in genome organization and stability, of the chloroplast genomes were low (37.3%) and were similar to that of other Asteraceae species, such as *Aster spathulifolius* Maxim. (37.28%) and *A. hypoleucus* (37.3%) (Ravi et al., 2008; Tyagi et al., 2019; Wang et al., 2019). We detected losses of the *trnT* (*GGU*) gene in *A. procerus* and *A. pycnophyllus*. In previous study, the loss of the tRNA was detected in some Asteraceae species (Lee et al., 2017).

### 4.2 Expansion and contraction of the border regions

The IR regions are known to be highly conserved in the genome of chloroplasts. During evolution, the expansion and contraction of the IR, LSC, and SSC regions are common, which leads to variability in genome length (Kim and Lee, 2004). In this study, the examination of chloroplast genome variation (**Figure 2**) showed that great expansion or contraction of the chloroplast IR region was not detected. However, *ycf1* and *ndhF* genes located at the SSC/IR border had the slight variation in position and length in the eight *Aster* chloroplast genomes, suggesting boundary contraction and expansion between the SSC/IR regions in *Aster* (Liu et al., 2018).

### 4.3 Repeat sequence analysis

The SSRs are effective molecular markers, and they are often used for species identification and population genetic analyses (Thiel et al., 2003). In the eight *Aster* species analyzed here, A/T repeats, AT/AT repeats, AAT/ATT repeats, and AAAT/ATTT repeats were commonly detected (**Figure 3C**). This phenomenon may be related to that the AT preference pattern is widely reported in many plant plastids (Somaratne et al., 2019). In the rearrangement of the complete chloroplast genomes and sequence divergence, larger and more complex repeat sequences may play an important role (Weng et al., 2014). The interspersed repeat sequences were more prevalent in the non-coding regions than the coding regions (Kim et al., 2015). In our study, the *ycf2* gene includes rich repeats, which contained many repeats: forward and palindromic. This result was consistent with the previous analysis that showed the gene has already been shown to be associated with many evolutionary events (Huang et al., 2010).

### 4.4 Sequence divergence and hotspots

DNA barcoding technology has been widely used in the species identification, phylogeny, and evolution (Doorduin et al., 2011; Palazzesi et al., 2022). In mVISTA analysis, the *matK*, *atpA*, *rps19*, *ycf2*, and *ycf1* genes had large differences and were putative markers for population genetic and barcoding analyses (**Figure 5**). Among these genes, the *matK* and *ycf1* genes have been used in previous plant phylogenetic and DNA barcoding analyses for land plants (Dong et al., 2015). Some regions of the plastomes of the eight *Aster* species showed high sequence divergence and might be used for phylogenetic reconstruction. However, these regions are different from the phylogenetic markers previously reported for Asteraceae (Do et al., 2019). Therefore, the complete chloroplast genome sequences and molecular markers might provide fundamental data for further studies on genus of *Aster* and related species in tribe Astereae.

### 4.5 Codon usage analysis

Thirty-one codons with RSCU value >1 were found, indicating that these codons are preferentially used in coding amino acids. An identical trend was discovered among the eight species (**Figure 7**). Leucine was the most abundant amino acid, whereas the cysteine was the least abundant amino acid, which is consistent with other Asteraceae species (Salih et al., 2017; Shen et al., 2017). In addition, most of A/U-ending codons had RSCU values >1; meanwhile, most of G/C-ending codons had RSCU values <1, indicating that amino acids tended to using A/U-ending codons, similar to a previous study (Zhao et al., 2021).

### 4.6 Phylogenetic analysis

In the tribe Astereae, there are numerous morphologically similar but distantly related taxa, such as some species of *Aster* (Noyes and Rieseberg, 1999). Whether some taxa should remain as genera or be merged into a single genus remains to be determined, such as genus *Kalimeris*, *Heteropappus*, and *Heteroplexis*, as wells as *Aster* series *Albescentes*, *Aster* Ser. *Hersileoides*, and *Aster* section *Alpigenia* (Nesom, 1994b; Noyes and Rieseberg, 1999; Li et al., 2012; Jafari et al., 2015; Korolyuk et al., 2015; Nesom, 2020). Nesom (1994b) proposed that *Aster* series *Albescentes* should be removed from *Aster*. In the Flora of China, *Aster* series *Albescentes* species *A. nitidus* and *A. hersileoides* were treated as the unplaced *Aster* group. The molecular phylogeny of Li et al. (2012) suggested that *Aster* section *Alpigenia* should be elevated to the new genera, series *Albescentes* is considered to be more closely related to section *Alpigenia*, and the *Aster* series *Hersileoides* is a well-supported monophyletic group. Therefore, according to the results of previous studies and the phylogenetic tree of this study, we classified the 25 species (not including outgroups) into five clades: clade A (core *Aster*), clade B (*Aster* series *Albescentes*), clade C (*Aster* Ser. *Hersileoides*), clade D (Alpine *Aster*, *Aster* section *Alpigenia*), and North American clade. Besides, the phylogenetic analysis of complete chloroplast genomes provided strong supports for these five clades (clade A, BS = 100; clade B, BS = 100; clade C, BS = 100; North American Clade, BS = 100; and clade D, BS = 100).

#### 4.6.1 Clade A (core *Aster*)

The eight species of *Aster* formed clade A with high support (**Figure 8**): *Aster tongolensis* Franch., *Aster souliei* Franch., *A. falcifolius*, *Aster tataricus* L.f., *A. pycnophyllus*, *Aster ageratoides* Turcz., *Aster fanjingshanicus* Y.L.Chen & D.J.Liu, and *A. spathulifolius*. Additionally, six species of the closely related genera *Kalimeris*, *Heteroplexis*, and *Heteropappus* were also included within clade A, supporting the placement of *Kalimeris*, *Heteroplexis*, and *Heteropappus* within *Aster*. The general characteristics of *Aster* are as follows: large herbs, leaves cauline, basal leaves, and proximal leaves withered at anthesis usually, stem leaves well developed, nearly as long as basal leaves, capitula many, much branched, in corymbiform, terminal solitary rarely, involucres herbaceous or membranous, involucres 3-numerous, unequal, imbricate, 2-3(7)–ribbed, and secretory cavity few.

In the previous studies, *Heteropappus* was considered for generic rank based on the heteromorphic pappus of ray and disc flowers (Jones, 1980; Noyes and Rieseberg, 1999; Chen et al., 2011; Jafari et al., 2015). Based on RFLPs and gene sequences, it is suggested that *Heteropappus altaicus* should be classified within *Aster* (Ito et al., 1998; Li et al., 2012). Our study showed that *Aster altaicus* Willd. (=*Heteropappus altaicus*) belong to clade A (BS = 100), supporting the previous results. Li et al. (2012) proposed *A. pycnophyllus* should be kept separate as it was found to be nested within a clade with *Myriactis* Less. and distantly related to *Aster*. In our study, the result shown that *A. pycnophyllus* was nested within clade A, with a strong support (BS = 97).

The genus *Kalimeris* is defined by the compressed obovoid-oblong of achenes and short lobe only comprising *K. indica* (Ito et al., 1995, Ito et al., 1998; Chen et al., 2011). These traits have also been analyzed in previous studies. However, many species of *Aster* also exhibit similar characteristics such as *Aster smithianus* Hand.-Mazz., *Aster souliei* Franch., and *Aster hunanensis* Hand.-Mazz. Hybridization between *Aster* and *Kalimeris* was also observed frequently (Tara, 1972; Gu and Hoch, 1997; Li et al., 2012). Gu and Hoch (1997) revised the genus *Kalimeris* based on morphological and cytological evidence showing a close phylogenetic relationship between *Kalimeris* and *Heteropappus*. Using RFLPs and DNA molecular markers, *Kalimeris* was shown to be not an independent genus and embedded within the genus *Aster* (Ito et al., 1995; Li et al., 2012). In our study (**Figure 8**), *Aster indicus* L. (=*Kalimeris indicus*), *Aster pekinensis* (Homce) F.H.Chen (=*Kalimeris pekinensis*), and *Aster procerus* (=*Kalimeris procerus*) fall within clade A (BS = 100), supporting the including of *Kalimeris* in *Aster*.

The genus *Heteroplexis*, comprising five species, is an herb endemic to Guangxi, China (Chen et al., 2011). In the Flora of China, the genus *Heteroplexis* shares similarities in morphology and inflorescence with the genus *Aster*, but they could be distinguished by its bilaterally symmetrical corolla and climbing or erect herb (Chen et al., 2011). Therefore, it is placed within the subtribe Asterinae as close allies of *Aster*. Based on the number of outer flowers over the number of bisexual flowers, Zhang and Bremer (1993) treated *Heteroplexis* in *Erigeron*-*Conyza* group. According to some characters, e.g., disciform capitula, oblong-obovoid achenes, and long corolla lobes, Nesom (1994a) treated *Heteroplexis* as a member of Baccharidinae. In recent study, it is the unplaced *Aster* group (Nesom and Robinson, 2007). Our results suggested that *Heteroplexis* should be included with *Aster* and treated as a synonym of *Aster*.

#### 4.6.2 Clade B (*Aster* series *Albescentes*)

The species of clade B exhibit a shrubby growth habit and are classified within the *Aster* series *Albescentes* (Li et al., 2012; Nesom, 2020). Based on the characters of muti-layers involucre and muti-ribs achene, Nesom (1994b) proposed that *Aster* series *Albescentes* has a distinct position in *Aster*. Based on the character of pappus, Nesom (1994b) noted that *A.* series *Albescentes* is sister to the NA *Doellingeria* Nees. In the Flora of China, the species of *Aster* ser. *Albescentes* were considered as the unplaced *Aster* group (Chen et al., 2011). Li et al. (2012) showed that ser. *Albescentes* is a monophyletic taxon with high support in a polytomy with *Myriactis* and other segregates of *Aster* s.s., implying that series *Albescentes* may belong to the Australasian lineages, in disagreement with the study of Nesom (1994b). In this study, *A. albescens*, *A. argyropholis*, *A. lavandulifolius*, *A. polius*, and *A. hypoleucus* formed a strong supported clade B (BS = 88/100) as sister of clade C (*Aster* Ser. *Hersileoides*) (**Figure 8**). It demonstrates that series *Albescentes* is a well-supported monophyletic genus. The newly defined taxon possesses the following distinct characteristics: shrubs, leaves cauline, basal leaves and proximal leaves withered at anthesis usually, non-rosulate, stem leaves well developed, nearly as long as basal leaves, capitula many, much branched, in corymbiform, terminal solitary rarely, involucres herbaceous or membranous, involucres 3-5, imbricate, margin membranous, irregularly lobed. margin membranous, irregularly lobed, and 4-5(8)–ribbed.

#### 4.6.3 Clade C (*Aster* Ser. *Hersileoides*)

*Aster* series *Hersileoides* consists of two species, *Aster hersileoides* C.K.Schneid. and *Aster nitidus* C.C.Chang (Yin et al., 2010; Chen et al., 2011). Chen et al. (2011) treated the *A. hersileoides* within the unplaced status in *Aster*. Based on molecular phylogenetic studies, Li et al. (2012) strongly suggested that *Aster* ser. *Hersileoides* should be removed from *Aster* and considered as a separate genus. Our results supported that the series (represented by *A. hersileoides*) should be kept separately from *Aster*. They are characterized by shrubs, leaves cauline, non-rosulate, leaf oblanceolate and glabrous, capitula many, terminal solitary, involucres 3-5, imbricate, 2 inner involucres equaling, and 3-ribbed. It is reasonable to propose the elevation of the *Aster* Series *Hersileoides* to a generic rank, considering its unique traits and the phylogenetic results here. Further investigations and comprehensive molecular analyses will be essential in demonstrating the full taxonomic status and evolutionary relationships of this clade.

#### 4.6.4 Clade D (Alpine *Aster*)

In our study, clade D contained *Aster batangensis* Bureau & Franch., *Aster flaccidus* Bunge, and *A. yunnanensis* (BS = 97). In previous study, these species have been placed in the genus *Aster* (Nesom, 1994b; Nesom and Robinson, 2007; Chen et al., 2011). Li et al. (2012) recognized that *A. batangensis* is closely allied with *Aster senecioides* Franch. and *Aster fuscescens* Bureau & Franch. from *Aster* section *Alpigenia*. However, based on morphologic differences, the study of Li et al. (2012) supported that *A. batangensis* might represent a monotypic genus. In our study, *A. batangensis* also has a distinct position in clade D. The clade has some distinctive characteristics: herbs dwarf, leaves rosulate, basal leaves at anthesis, cauline leaves reduced, significantly shorter than basal leaves, capitula solitary few, scapose, rarely branched, involucres herbaceous 2-3, subequal, non-imbricate, 3-4(6), secretory cavity, and secretory cavity few. Our molecular findings strongly support that clade D is an independent group.

In conclusion, the previously classification and definition of *Aster* is not monophyletic. Clade A (core *Aster*) includes most *Aster* taxa, *Heteropappus*, *Kalimeris*, and *Heteroplexis*. Additionally, clade B (*Aster* series *Albescentes*), clade C (*Aster* Ser*. Hersileoides*), and clade D (Alpine *Aster*) are identified as independent groups. Furthermore, it is estimated that the genus *Aster* comprise more than 152 species. However, this study only encompassed 25 species and two outgroup species. Therefore, a more comprehensive and extensive sampling of chloroplast genome and more data are necessary to conduct a thorough and comprehensive phylogenetic study of the genus *Aster* and its related genera. Based on the results of both general morphological and molecular phylogenetic analysis, the identification key was presented as following.

Key to the *Aster* and related species (clades A to D):

1. herbs, achenes 2-3(7)–ribbed, phyllaries 2-numerous-layers
2. herbs large or occasionally dwarf, achenes 2-3(7)–ribbed, phyllaries 3-numerous-layers……………………………………………………………….Clade A (core genus *Aster*)
2. herbs dwarf or occasionally large few, achenes 3-4(6)–ribbed, phyllaries 2-3-layers…………………………………………………………………………………………………………………………Clade D (alpine *Aster*)
1. shrubs, achenes (3)5-7-ribbed, phyllaries 3-numerous-layers
3. achenes 4-5(8)–ribbed………Clade B (*Aster* ser. *Albescentes*)
3. achenes 3 ribbed……….Clade C (*Aster* ser. *Hersileoides*)

### 4.7 Divergence time estimations

The divergence time estimation of *Aster* relied on secondary calibration because of the lack of fossil record for most *Aster* taxa. Most species of *Aster* and its related genera are distributed in East Asia (Nesom, 1994b; Brouillet et al., 2009; Chen et al., 2011). The result of Brouillet et al. (2009) indicated that *Aster* originated from a clade with a dispersal from Australasia into East Asia. The result of our molecular dating suggests that clades A and C began to diversify in the late Oligocene (23.15 Mya and 24.29Mya, respectively). Clades B and D originated in the Early Miocene (15.13 Mya and 21.66Mya, respectively). The rapid radiation may be related to collisions between geological plates (Audley-Charles, 1987; Liu et al., 2002). Geologic uplift events (first of which began at about 50 Ma) have taken place in the Tibetan Plateau during at least four different periods since the early Miocene, i.e., 22 Mya, 15–13 Mya, 8–7 Mya, and 3.5–1.6 Mya (Shi et al., 1998; Guo et al., 2002; Spicer et al., 2003). The origin of the four clades (Clade A-D) likely occurred independently at first two stages of the uplift and formation of the Tibetan Plateau. Geological evidence suggests that the strong uplift of the Tibetan Plateau, coupled with favorable oceanic and continental environments, produced a strong Asian monsoon dominated by the Summer Monsoon (Shi et al., 1998; Ding et al., 2020). During this uplift movement, the original Planetary Wind System in East Asia was changed and the arid zone retreated to the northwest (Shi et al., 1998; Ding et al., 2020). Eastern China was gradually covered by tropical or subtropical forests. This scenario also correlates with the current habitat preferences of the studied taxa (clade A, understorey vegetation; clades B and C, dry slopes and scree regions; and clade D, cold and dry alpine meadows). The similar rapid radiation has also been found in other groups of Asteraceae in the Tibetan Plateau, such as *Saussurea* (Liu et al., 2002) and the *Dolomiaea*-*Diplazoptilon*-*Xanthopappus* group (Wang et al., 2007).

## 5 Conclusions

The complete chloroplast genomes of the eight *Aster* species were sequenced in this study. The results revealed that cp genome size, structure, gene content, as well as compositional organization were highly conserved among these species. The chloroplast genomes of all species exhibited the standard quadripartite structure, and the size of these species of *Aster* varied from 152,045 bp to 152,729 bp. They include 87 protein-coding genes, 37/38 tRNA genes, and eight rRNA genes. They have three/four types of repeats, and the number of SSRs ranged from 75 to 99. Genes located at the junctions were well conserved among the *Aster* species. Furthermore, the genic and IR regions were more conserved than the intergenic and SC regions, respectively. In addition, the plastid genome structure of *Aster* exhibited high consistency and was obviously different in some regions, such as *rps19*, *ycf1*, and *ndhf*. Furthermore, the preferences for codon use in our study are all similar. The most prevalent amino acid was leucine, whereas the rarest one was cysteine. Moreover, we detected six hotspots that could be used as candidate DNA barcodes. The analysis of complete chloroplast genomes and combined datasets provided clear evidence supporting the moderate to strong differentiation of clades (clades A, B, C, and D and North American clade). The phylogenetic results showed that the traditionally defined *Aster* was not monophyly. For the delimitation of the genus *Aster*, *Kalimeris*, *Heteropappus*, and *Heteroplexis*, the closed allied genera of *Aster* were revealed to be nested within the *Aster* clade and should be included in *Aster*. Additionally, we suggest that the clade B (*Aster* series *Albescentes*), clade C (*Aster* Ser*. Hersileoides*), and clade D (Alpine *Aster*) should be treated as separated genera and taxonomic treatment. Divergent time estimate showed that the divergent time of clade A was dated back to 23.15Mya. Clades B, C, and D were divergent from 15.13 Mya, 24.29 Mya, and 21.66Mya, respectively. Our analyses suggested that the divergence of the genus *Aster* is closely related to the uplift of the Qinghai-Tibet Plateau. This study sequenced eight plastid genomes of *Aster*, provided a well resolved phylogenetic tree of *Aster* and related genera, and selected putative markers for further barcoding analysis. This study is important for us to understand the phylogeny and evolution of *Aster* and the further phylogenetic, population genetic, and related studies.

## Data availability statement

The datasets presented in this study can be found in online repositories. The names of the repository/repositories and accession number(s) can be found below: https://www.ncbi.nlm.nih.gov/, OM912721; https://www.ncbi.nlm.nih.gov/, ON515470; https://www.ncbi.nlm.nih.gov/, ON515468; https://www.ncbi.nlm.nih.gov/, ON515469; https://www.ncbi.nlm.nih.gov/, ON515467; https://www.ncbi.nlm.nih.gov/, OM912720; https://www.ncbi.nlm.nih.gov/, OM912719; https://www.ncbi.nlm.nih.gov/, OM912718.

## Author contributions

HC: Writing – original draft, Conceptualization. TL: Writing – original draft, Software, Methodology, Formal analysis. XC: Writing – original draft, Conceptualization. TQ: Writing – review & editing, Software, Methodology, Formal analysis. XZ: Writing – review & editing, Software, Methodology, Formal analysis. JL: Writing – original draft, Conceptualization. BL: Writing – review & editing, Supervision. GZ: Writing – review & editing, Supervision. ZF: Writing – review & editing, Supervision, Resources.
